# Coexpression analysis of lncRNAs and mRNAs identifies potential regulatory long noncoding RNAs involved in the inflammatory effects of lipopolysaccharide on bovine mammary epithelial cells

**DOI:** 10.1186/s12917-023-03780-4

**Published:** 2023-10-17

**Authors:** Xiaojing Xia, Jie Hou, Pengfei Ren, Mingcheng Liu, Lei Wang, Xiaobing Wei, Zhanwei Teng, Oksana Kasianenko, Likun Cheng, Jianhe Hu

**Affiliations:** 1 College of Animal Science and Veterinary Medicine, Henan Institute of Science and Technology, Xinxiang, 453003 PR China; 2 Faculty of Veterinary Medicine, Sumy National Agrarian University, Sumy, Ukraine; 3 Shandong Binzhou Animal Science and Veterinary Medicine Academy, Binzhou, 256600 PR China

**Keywords:** Mastitis, lncRNA, mRNA, Mammary gland epithelial cells, RNA-seq

## Abstract

**Background:**

The infection of bovine mammary glands by pathogenic microorganisms not only causes animal distress but also greatly limits the development of the dairy industry and animal husbandry. A deeper understanding of the host’s initial response to infection may increase the accuracy of selecting drug-resistant animals or facilitate the development of new preventive or therapeutic intervention strategies. In addition to their functions of milk synthesis and secretion, bovine mammary epithelial cells (BMECs) play an irreplaceable role in the innate immune response. To better understand this process, the current study identified differentially expressed long noncoding lncRNAs (DE lncRNAs) and mRNAs (DE mRNAs) in BMECs exposed to *Escherichia coli* lipopolysaccharide (LPS) and further explored the functions and interactions of these lncRNAs and mRNAs.

**Results:**

In this study, transcriptome analysis was performed by RNA sequencing (RNA-seq), and the functions of the DE mRNAs and DE lncRNAs were predicted by Gene Ontology (GO) and Kyoto Encyclopedia of Genes and Genomes (KEGG) pathway enrichment analyses. Next, we constructed a modulation network to gain a deeper understanding of the interactions and roles of these lncRNAs and mRNAs in the context of LPS-induced inflammation. A total of 231 DE lncRNAs and 892 DE mRNAs were identified. Functional enrichment analysis revealed that pathways related to inflammation and the immune response were markedly enriched in the DE genes. In addition, research results have shown that cell death mechanisms, such as necroptosis and pyroptosis, may play key roles in LPS-induced inflammation.

**Conclusions:**

In summary, the current study identified DE lncRNAs and mRNAs and predicted the signaling pathways and biological processes involved in the inflammatory response of BMECs that might become candidate therapeutic and prognostic targets for mastitis. This study also revealed several possible pathogenic mechanisms of mastitis.

## Background

Mastitis constitutes a series of inflammatory reactions in the mammary glands of cows due to stimulation by various physical factors, chemical factors, pathogenic microorganisms, and other factors, and it affects the lactation function of dairy cows, leading to decreases in milk yield and quality and hindering the development of the dairy industry and animal husbandry [1]. More than 130 kinds of pathogenic microorganisms, including *Escherichia coli* (*E. coli*), *Streptococcus uberis* (*S. uberis*), *Streptococcus agalactiae* (*S. agalactiae*), and *Staphylococcus aureus* (*S. aureus*), can cause mastitis in dairy cows [2]. *E. coli* is one of the most common pathogens causing mastitis in dairy cows [3], and its pathogenicity is due to several virulence factors, such as toxins, adhesins, hemolysins, and lipopolysaccharide (LPS) [4]. Stimulation by *E. coli* LPS can alter the immune response in cows, leading to changes in the relative expression of IFN-γ and IL-10 in peripheral blood mononuclear cells [5, 6]. LPS, the main microbe-associated molecular pattern (MAMP) of *E. coli*, can trigger the innate immune response in the mammary gland by binding to components of the Toll-like receptor (TLR) signaling pathway and NOD-like receptor (NLR) signaling pathway. This process leads to activation of the NF-κB signaling pathway and induction of IL-8, IL-6, IL-1β, and TNF-α expression, as well as inhibition of casein synthesis, which is considered the main virulence factor promoting the pathogenicity of *E. coli* [7–9].

RNA sequencing (RNA-seq) is a highly effective method for exploring the molecular mechanisms associated with disease occurrence, as it can be used to study gene expression and function to reveal specific physiological/pathological processes. In addition to mRNAs, long noncoding RNAs (lncRNAs) have also attracted increasing research attention in recent years [10]. Numerous studies have shown that lncRNAs are involved in the expression of various inflammatory genes and the activation of signaling pathways [11, 12]. For example, the lncRNA Mirt2 is a negative feedback regulator of excessive inflammatory responses [13], and the lncRNA Carlr can interact with the activated NF-κB transcription factor complex [14]. Moreover, lncRNAs can play biological roles in transcriptional regulation, epigenetic modification, and posttranscriptional regulation and regulate the occurrence and development of diverse animal and human diseases, including mastitis in dairy cows [15–17]. However, the effects of lncRNA expression profiles and mRNA‒lncRNA regulatory networks in mastitis remain largely unexplored.

Bovine mammary gland epithelial cells (BMECs) are the initial site of the intersection of bacterial infection and mammary immunity. At this initial junction, BMECs not only function as a natural barrier to the invasion of pathogens but also trigger the host’s earliest immune recognition and immune response to pathogenic microorganisms and play an important role in coordinating the expression of downstream immune molecules and the response of immune cells [18]. To investigate the molecular regulatory mechanism of mastitis in dairy cows and to identify potential prevention and treatment targets, we performed transcriptome analysis by RNA-seq to construct the expression profiles of differentially expressed lncRNAs (DE lncRNAs) and mRNAs (DE mRNAs) in LPS-stimulated mammary gland epithelial cells from dairy cows. We also constructed a coexpression network to reveal the potential roles of these lncRNAs and mRNAs, and their interactions during mastitis in dairy cows. The current study provides a theoretical foundation for the application of lncRNAs and mRNAs in dairy cow breeding.

## Results

### Sequencing data quality assessment and comparison

The denaturing agarose gel electrophoresis results showed that the total RNA quality of all samples in the LPS group and the control group was high, and no genomic contamination or degradation was detected. All OD_260/280_ ratios were between 1.8 and 2.0, and the total RNA concentration, integrity and purity met the criteria for sequencing. The results of sequencing quality control showed that the number of raw reads in the 6 samples ranged from 70,590,842 to 101,152,596 (Table 1). After removing low-quality raw reads, between 70,583,830 and 101,145,294 clean reads were obtained. The clean ratio (the proportion of clean reads to raw reads) was greater than 99.98%, and the Q30 value exceeded 89.00%, which comprehensively indicated that the sequencing results were suitable for downstream analysis and that the data volume was high.

### Differential expression profile analysis and verification of lncRNAs and mRNAs

**Fig. 1:**
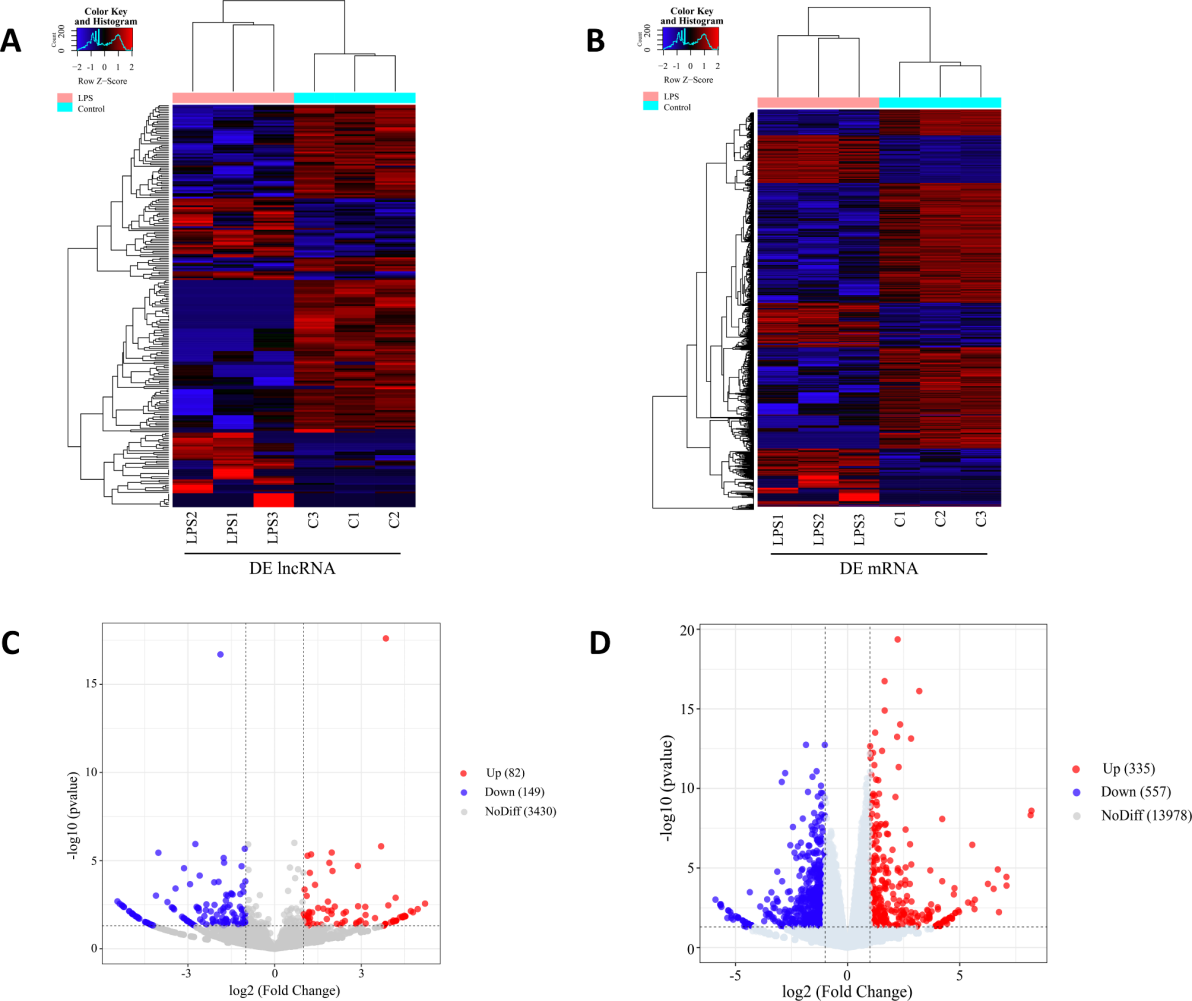
DE lncRNA and mRNA expression profiles in the LPS group and the control group. (**A**) Hierarchical clustering of DE lncRNAs; (**B**) Hierarchical clustering of DE mRNAs; (**C**) Volcano plot of DE lncRNAs; (**D**) Volcano plot of DE mRNAs. DE, differentially expressed

**Fig. 2:**
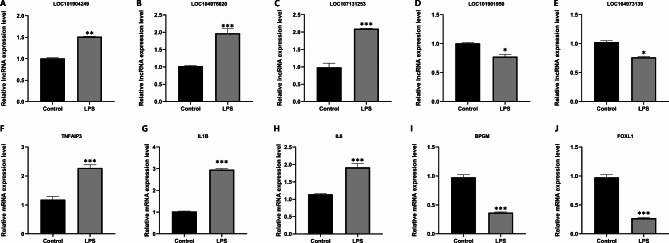
Validation of RNA-seq data using RT‒qPCR. *P < 0.05; **P < 0.01; ***P < 0.001

A total of 3661 lncRNAs and 14,870 mRNAs were identified between the control group and LPS group in this experiment. The heatmaps of the DE lncRNAs and DE mRNAs show that the expression of many lncRNAs and mRNAs in the LPS group was altered compared with that in the control group (Fig. 1A, B). The volcano plot shows that 231 DE lncRNAs (82 upregulated and 149 downregulated, Fig. 1C) and 892 DE mRNAs (335 upregulated and 557 downregulated, Fig. 1D) were identified in this experiment. Table 2 lists the 10 mRNAs and 10 lncRNAs with the most significant differential expression. The qRT‒PCR results showed that the expression trends of the selected DE lncRNAs and DE mRNAs were consistent with those identified by RNA-seq (Fig. 2), confirming that the RNA-seq data were reliable.

**Table 1:** Data information of all samples collected by RNA-seq

Sample	Raw Reads	Clean Reads	Clean Ratio	Q30
C1	85,912,138	85,906,558	99.9935%	90.14%
C2	86,038,248	86,033,092	99.9940%	89.70%
C3	101,152,596	101,145,294	99.9928%	90.32%
LPS1	70,590,842	70,583,830	99.9901%	90.19%
LPS2	74,915,290	74,904,990	99.9863%	90.04%
LPS3	76,226,896	76,219,704	99.9906%	90.31%

### Prediction of target genes of the DE lncRNAs

An important function of lncRNAs is regulating the expression of adjacent protein-coding genes. Potential target genes for cis- and trans-regulation are predicted based on their positional relationships and sequence similarity. The prediction results showed that the 231 DE lncRNAs targeted 145 mRNAs, among which 10 were also among the 892 DE mRNAs. Table 3 lists the top 10 DE lncRNAs and the predicted target genes that they differentially regulate.

**Table 2:** The top 10 differentially expressed lncRNA and mRNA

Gene name	Log_2_FC	P Value	Gene type
*LOC107133214*	-5.440920105	0.002056876	lncRNA
*LOC101906469*	-5.36371342	0.002798547	lncRNA
*LOC101906880*	-5.289109266	0.003416971	lncRNA
*LOC112445200*	-5.267274784	0.003619504	lncRNA
*LOC104974443*	-5.227620991	0.00419241	lncRNA
*LOC104976078*	-5.216318697	0.004313335	lncRNA
*LOC112443173*	5.208187098	0.00274938	lncRNA
*LOC107132924*	-5.03318542	0.007388175	lncRNA
*LOC101905883*	-5.03318542	0.007388175	lncRNA
*LOC112443858*	4.954749154	0.005623408	lncRNA
*POU3F3*	8.202188286	2.54029E-09	mRNA
*HOXD8*	8.165331514	4.75853E-09	mRNA
*PPARGC1A*	7.090106376	0.000128232	mRNA
*HOXD10*	7.086571341	3.62166E-05	mRNA
*HSPA6*	6.752952915	0.005929194	mRNA
*DACH1*	6.699586319	1.23994E-05	mRNA
*PAX2*	6.515770426	0.000203191	mRNA
*CDH11*	6.25948693	9.96586E-05	mRNA
*BARX2*	-5.89620123	0.000968371	mRNA
*LOC112445942*	-5.684179222	0.001927003	mRNA

### Gene Ontology (GO) and Kyoto Encyclopedia of Genes and Genomes (KEGG) enrichment analyses of the DE genes

**Fig. 3:**
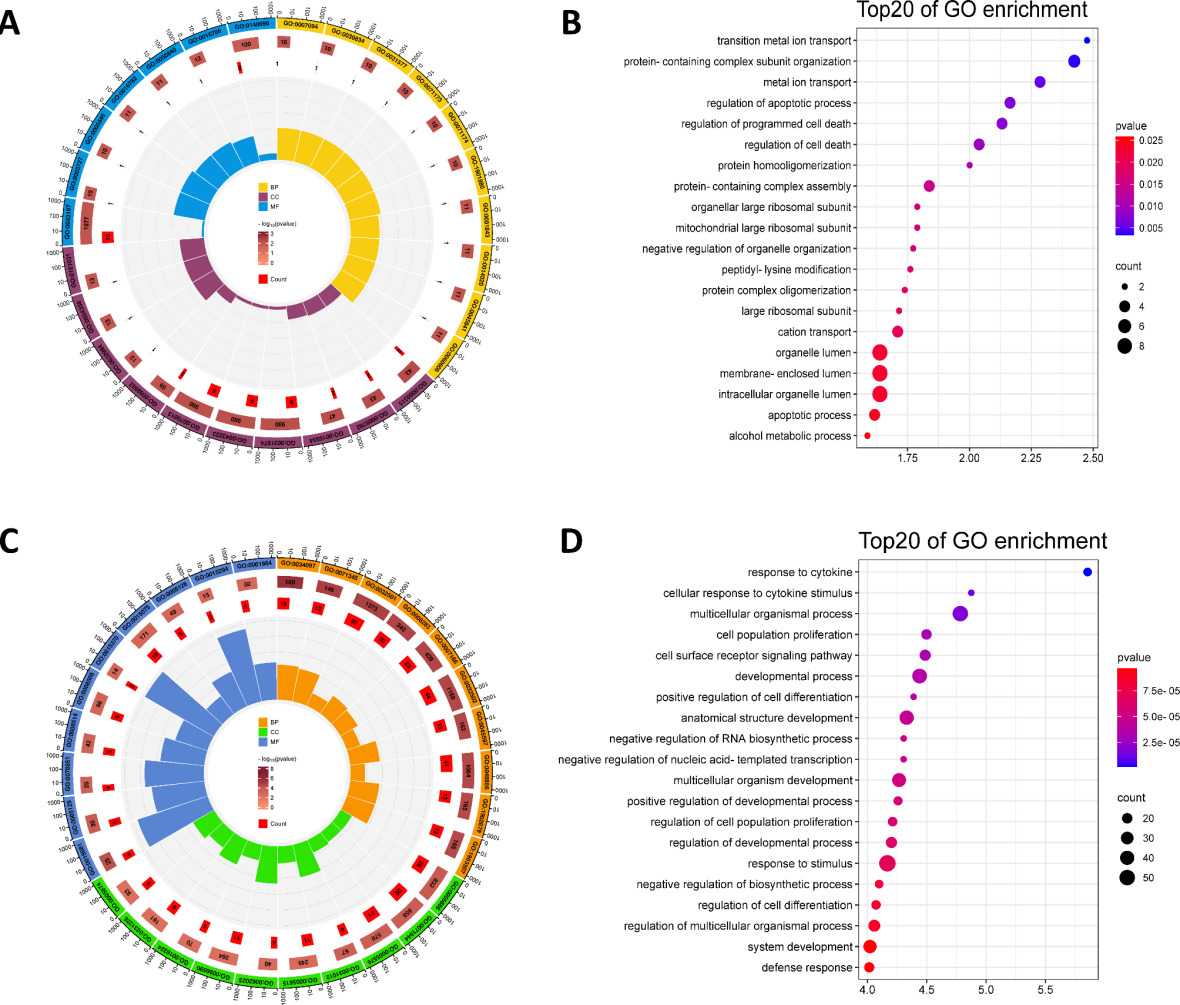
GO enrichment analysis of the target genes of the DE lncRNAs and the DE mRNAs. (**A**) The top 10 biological process, cellular component and molecular function GO terms enriched in the target genes of the DE lncRNAs; (**B**) The top 20 GO terms enriched in the target genes of the DE lncRNAs; (**C**) The top 10 GO biological process, cellular component and molecular function terms enriched in the DE mRNAs; (**D**) The top 20 GO terms enriched in the DE mRNAs

**Fig. 4:**
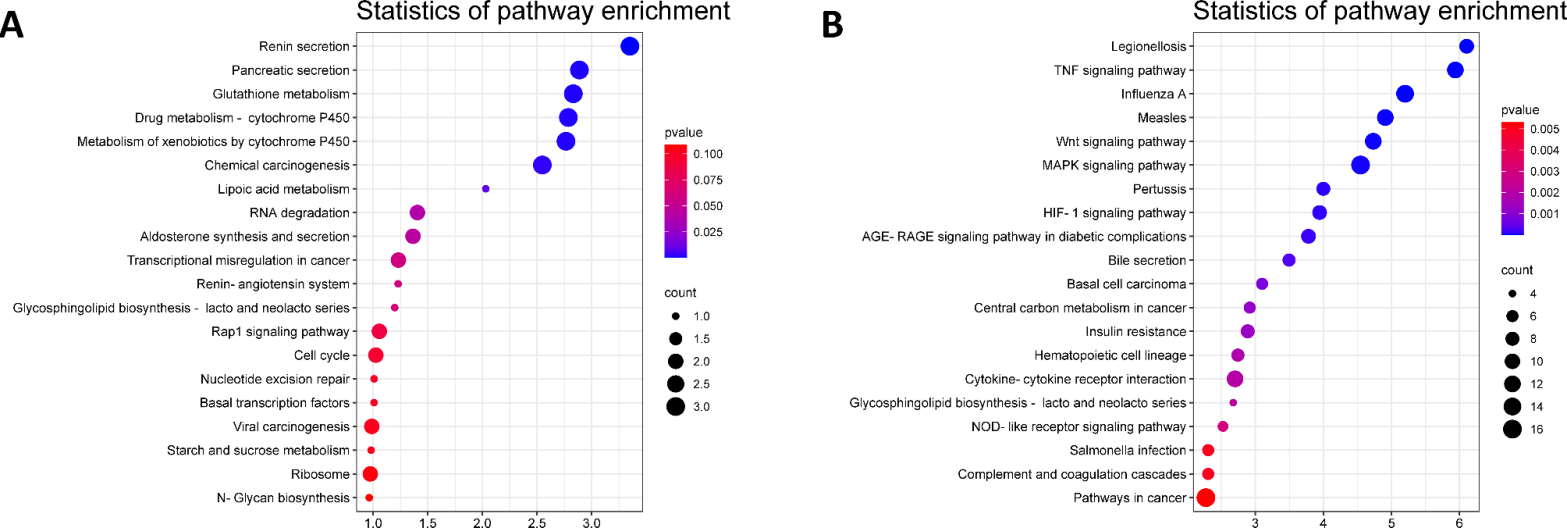
KEGG enrichment analysis of the target genes of the DE lncRNAs (**A**) and the DE mRNAs (**B**)

To further investigate the biological functions of the DE genes, the target genes of the DE lncRNAs and the DE mRNAs were functionally annotated and subjected to enrichment analysis, and their potential functions were suggested. A total of 95 biological functions and 44 pathways in the GO and KEGG databases, respectively, were significantly enriched in the target genes of the DE lncRNAs. Figure 3 A shows the 10 GO terms with the highest enrichment scores in the molecular function, cellular component, and biological process categories. The top 20 GO terms among all GO terms sorted by enrichment score are presented in Fig. 3B. KEGG enrichment analysis showed the greatest enrichment of the target genes of the DE lncRNAs in the RNA degradation, cell cycle, Rap1 signaling pathway, NLR signaling pathway, and FcγR-mediated phagocytosis pathways, among other pathways. The signaling pathways with the top 20 enrichment scores are shown in Fig. 4A.

A total of 577 GO terms and 75 KEGG pathways were significantly enriched in the DE mRNAs. Figure 3 C shows the 10 GO terms with the highest enrichment scores in the molecular function, cellular component, and biological process categories. The top 20 GO terms among all GO terms sorted by enrichment score are shown in Fig. 3D. KEGG enrichment analysis showed the greatest enrichment of the DE mRNAs in the following pathways: TNF signaling pathway, NLR signaling pathway, cytokine‒cytokine receptor interaction, HIF-1 signaling pathway and MAPK signaling pathway. The signaling pathways with the top 20 enrichment scores are shown in Fig. 4B.

**Table 3:** Top 10 up and down regulation lncRNA and their predicted target genes

lncRNA	Log_2_FC	P Value	Associated gene name
*LOC112443858*	4.9547492	0.0056234	*NUDT3*
*LOC112441812*	4.7165943	0.007275	*MFSD4A*
*LOC101901948*	4.520337	0.0145542	*RBM47*
*LOC101902135*	4.5134837	0.0146858	*PSMG2*
*LOC112447298*	4.4374993	0.0163349	*PHLDB2*
*LOC101902838*	4.416177	0.0148463	*LOC101902838*
*LOC100300632*	4.2073888	0.0256199	*NYX*
*LOC100847780*	4.1959875	0.0259524	*ELP2*
*LOC101907342*	4.1887657	0.0012832	*IGF1R*
*LOC112447494*	4.0185917	0.0319697	*LNPEP*
*LOC107133214*	-5.440920105	0.002057	*FMOD*
*LOC101906469*	-5.36371342	0.002799	*MYOZ2*
*LOC101906469*	-5.36371342	0.003417	*SYNPO2*
*LOC101906880*	-5.289109266	0.00362	*USP50*
*LOC112445200*	-5.267274784	0.00419241	*LOC100297779*
*LOC104974443*	-5.227620991	0.0043133	*SPSB1*
*LOC107132924*	-5.03318542	0.007388	*PNPT1*
*LOC107132924*	-5.03318542	0.007388	*PPP4R3B*
*LOC107132924*	-5.03318542	0.01033648	*PPP4R3B*
*LOC101905883*	-5.03318542	0.00973874	*TMEM139*

### LncRNA–mRNA coexpression analysis

**Fig. 5:**
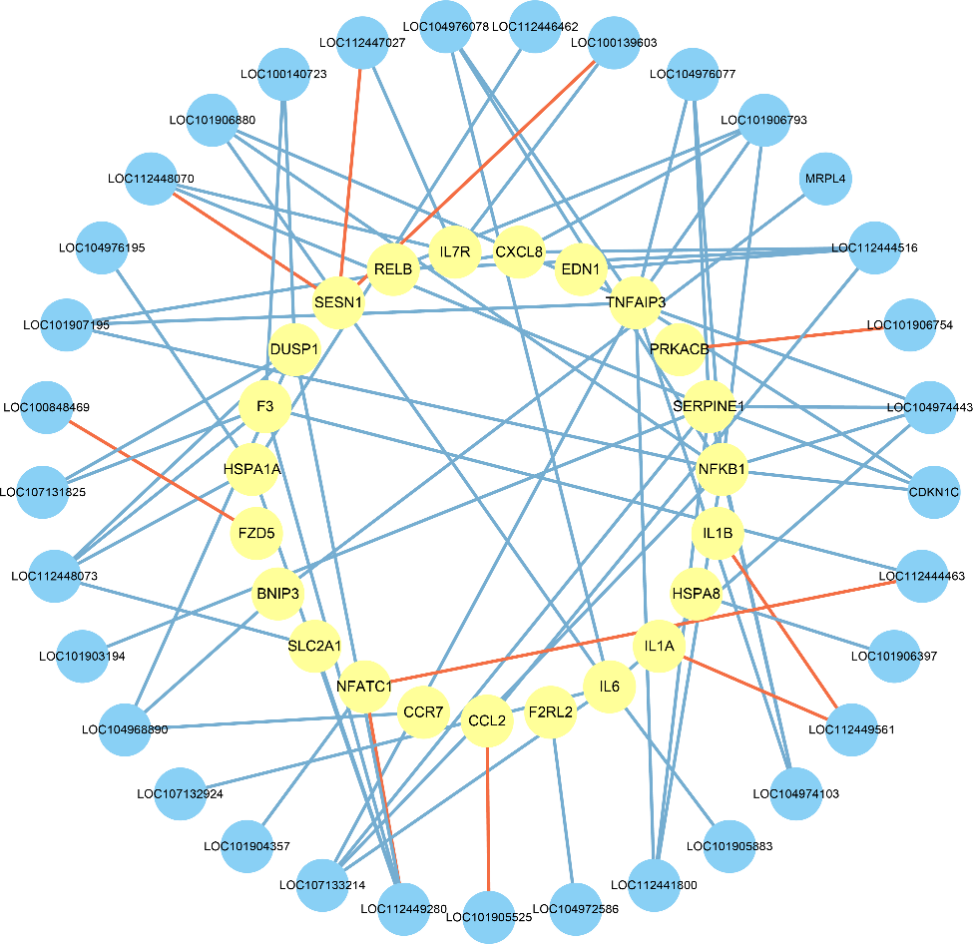
Coexpression network diagram of the inflammation-related mRNAs and lncRNAs

**Fig. 6:**
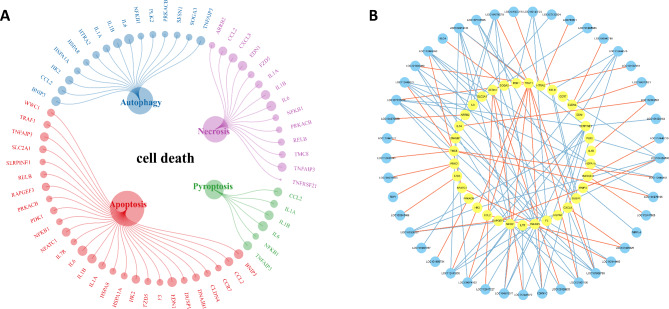
DE mRNAs and lncRNAs involved in cell death in LPS-treated bovine mammary epithelial cells. (**A**) The list of DE mRNAs related to cell death; (**B**) Coexpression network diagram of the cell death-related mRNAs and lncRNAs

To further explore the potential roles of the lncRNAs, 23 DE mRNAs related to inflammation, as indicated by the GO annotations in the AmiGO 2 project and KEGG database, were selected by screening. A total of 33 lncRNAs with strong correlations (|R| ≥ 0.99, P < 0.05) were identified by correlation analysis between these DE mRNAs and the DE lncRNAs. Ten pairs of positive correlations and 66 pairs of negative correlations were identified (Fig. 5). Lytic cell death is a key component of the host immune response, and when this process is dysregulated, the large number of dead cells can trigger inflammation and aggravate inflammatory diseases. In this study, 21 mRNAs were found to be associated with inflammatory cell death (necroptosis or pyroptosis). It is speculated that in addition to inflammation, inflammatory cell death may play a profound role in the development of mastitis. We summarized the DE mRNAs related to different types of cell death and analyzed the correlations between these genes and the DE lncRNAs. A total of 35 cell death-related genes were identified and included 27 apoptosis-related genes, 14 necroptosis-related genes, 6 pyroptosis-related genes, and 15 autophagy-related genes (Fig. 6A). A total of 39 lncRNAs strongly correlated with the cell death-related mRNAs (|R| ≥ 0.90, P < 0.05) were identified by correlation analysis between these mRNAs and the DE lncRNAs. Forty-four pairs of positive correlations and 77 pairs of negative correlations were identified (Fig. 6B).

## Discussion

Mastitis is a common inflammatory disorder caused by pathogenic infection, trauma and other factors; it is difficult to treat, not only endangering the health of cows but also affecting the quality and safety of dairy products [3]. Research has shown that lncRNAs play important modulatory roles in the occurrence and development of various inflammatory diseases [19]. However, to date, only a few researchers have used RNA-seq analysis to explore the roles of DE lncRNAs in mastitis in dairy cows. Ozdemir et al. identified 392 new lncRNAs and 57 DE lncRNAs in the mammary gland tissues of cows infected with *Mycobacterium bovis*, and the target genes of these DE lncRNAs are involved in the regulation of signaling pathways such as the PI3K-Akt, NF-κB and mTOR pathways [20]. Studies have shown that the aforementioned signaling pathways regulate cytokine production, the inflammatory response, apoptosis and immunity are closely associated with the occurrence and development of mastitis [21, 22]. Chen et al. found that the target genes of the DE lncRNAs in mastitis in dairy cows were clustered in signaling pathways such as the TNF, Notch, MAPK, Hippo and p53 pathways, which are involved in bacterial invasion and adhesion, oxidative stress and inflammation [23]. Studies have suggested that lncRNAs may regulate the progression of mastitis in dairy cows by affecting the expression of genes involved in pathways associated with inflammation, apoptosis and autophagy [20–24]. In this study, high-throughput sequencing was used to analyze the changes in the mRNA and lncRNA expression profiles in dairy cows with LPS-induced mastitis, 231 DE lncRNAs and 892 DE mRNAs were identified.

To elucidate the mechanism and molecular regulatory network of lncRNA-mediated regulation of bovine mastitis, target genes of the DE lncRNAs were predicted and subjected to functional analysis. The results indicated that the target genes of the DE lncRNAs were involved in pyroptosis, necroptosis, apoptosis and signaling pathways related to immune response regulation. Studies have shown that the Rap1 signaling pathway, NLR signaling pathway and FcγR-mediated phagocytosis are closely related to inflammation [25]. The Rap1 signaling pathway plays an important role in inflammation, regulating multiple functions of inflammatory cells and intracellular signal transduction [26]. FcγR-mediated phagocytosis is essential for humoral and innate immune responses to infection and chronic inflammation [27]. Previous studies have confirmed that lncRNAs can modulate inflammation and cell death by regulating the expression of genes in the NOD-like receptor signaling pathway. For example, *lncRNA SNHG1* can act as a competitive endogenous RNA of *miR-7* to regulate the expression of NOD-like receptor family pyrin domain containing 3 (NLRP3), thereby activating the NLRP3 inflammasome signaling pathway [28]. *LINC00917* regulates the inflammation, proliferation and pyroptosis of nucleus pulposus cells by targeting the *miR-149-5p*/NLRP1 axis [29]. In addition, the enrichment scores of the target genes of the DE lncRNAs revealed high enrichment in signaling pathways related to renin secretion, the renin-angiotensin system (RAS) and aldosterone synthesis and secretion. The RAS regulates the blood pressure homeostasis and humoral and inflammatory responses through the ACE/AngII/AT1R and ACE2/Ang1-7/Mas axes and plays a key role in the regulation of the inflammatory response. Previous studies have found that the ACE/AngII/AT1R and ACE2/Ang1-7/MasR axes are involved in the regulation of inflammatory injury induced in mouse mammary epithelial cells by exposure to high concentrations of LPS [30]. In summary, we speculate that lncRNAs related to signaling pathways such as the Rap1 signaling pathway, NOD-like receptor signaling pathway, and FcγR-mediated phagocytosis are involved in the inflammatory response process to regulate the progression of mastitis in dairy cows.

In addition, we analyzed the functions of the DE mRNAs. The DE mRNAs may be involved in biological functions and signaling pathways related to apoptosis, pyroptosis, necroptosis and other cell death processes, as well as immune response regulation. Wei et al. found that histone-induced apoptosis, necrosis and pyroptosis were associated with mastitis-related bovine mammary epithelial cell injury in an in vitro experiment [31]. Unlike accidental cell death, regulated cell death (RCD) can be mediated through a series of molecular mechanisms and signaling pathways [32]. The most widely studied form of RCD is apoptosis, which is triggered mainly by the activation of caspase family proteases [32]. Signaling pathways such as the TNF, MAPK, HIF-1, and NOD-like receptor pathways and cytokine‒cytokine receptor interactions have been shown to be closely related to or associated with inflammation [33–35]. Interleukin genes play important roles in inflammation and the immune system, and some of these genes, such as IL-6, have been found to regulate the immune response in mastitis and have been identified as candidate biomarkers for subclinical mastitis [36]. *Zophobas morio* (*Z. morio*) hemolymph can decrease the levels of NLRP3, caspase-1 and NLRP6 and inhibit the secretion of IL-1β and IL-18, thereby attenuating *E. coli-* or *Staphylococcus simulans* (*S. simulans*)*-*induced pyroptosis; thus, it can be a new therapeutic candidate for bovine mastitis [37]. Li et al. reported that *E. coli* infection induced NLRP3 inflammasome assembly, caspase-1 activation, mitochondrial damage and increased reactive oxygen species (ROS) production, causing BMEC apoptosis, whereas *Lactobacillus rhamnosus* GR-1 inhibited the effect of *E. coli* and had a preventive and protective effect against *E. coli-*induced mastitis [38]. Our study showed that LPS treatment resulted in significant changes in the expression profiles of lncRNAs and mRNAs in bovine mammary epithelial cells and that terms and pathways related to cell death processes such as apoptosis and pyroptosis, as well as inflammation, were significantly enriched, indicating that lncRNAs and mRNAs play key roles in the inflammatory response of mammary epithelial cells.

NFKB1 and TNFAIP3 are well-known inflammatory markers that can mediate cell death programs and inflammatory responses, including apoptosis, autophagy, and pyroptosis [39–41]. The NF-κB signaling pathway plays a key role in inflammation, especially in mastitis [42]. Wu et al. showed that the activation of the NF-κB signaling pathway mediated by TLR4 is related to inflammation and innate immune responses during mastitis in dairy cows [43]. Akhtar et al. found that the protein and mRNA levels of proinflammatory cytokines (IL-6, IL-1β and TNF-α) in infected tissues were increased compared with those in uninfected tissues and that activation of the NF-κB/MAPK signaling pathway through TLRs further triggered mastitis-related gene expression, followed by activation of the innate immune response [44]. CCL2, CXCL1 and CXCL12 are chemokines related to chemotaxis and inflammation, and CCL2 can regulate the recruitment and polarization of macrophages. Increased secretion of *SERPINE1* is associated with inflammation and physical damage, and studies have shown that *miR-1275* targeting of *SERPINE1* can inhibit the proliferation, invasion and migration of glioma cells while promoting apoptosis [45, 46]. Our coexpression analysis showed that *LOC107133214* and *LOC104974443* could regulate the expression of *IL-6*, *NFKB1*, *SERPINE1* and *TNFAIP3*; *LOC112449280* and *LOC112448073* could regulate the expression of *DUSP1*, *F3*, *HSPA1A*, and *SLC2A1*; *LOC112449280* could regulate the expression of *NFATC1*; *LOC101906793* could regulate the expression of *CXCL8*, *NFKB1*, *RELB* and *TNFAIP3*; and *LOC112444516* could regulate the expression of *CCL2*, *CXCL8*, *EDN1* and *RELB*.

## Conclusion

In this study, the changes in the lncRNA and mRNA expression profiles in the LPS-induced inflammatory response model in BMECs were investigated. A total of 231 DE lncRNAs and 892 DE mRNAs were identified. These DE lncRNAs and DE mRNAs may be involved in the inflammatory response, pyroptosis, and apoptosis through various signaling pathways. LncRNAs such as *LOC107133214*, *LOC104974443* and *LOC101906793* can regulate the expression of inflammatory and cell death-related mRNAs such as *IL-6*, *NFKB1* and *TNFAIP3* and participate in the process of mastitis through NOD-like receptor, TNF, MAPK and other signaling pathways. This study provides new ideas for further revealing the pathogenesis of mastitis in dairy cows and identifies potential new targets and pathways for subsequent research on and treatment of mastitis in dairy cows.

## Methods

### Cell culture

The bovine mammary epithelial cell line MAC-T was obtained from the College of Animal Science and Technology of Henan Institute of Science and Technology. The cells were cultured in DMEM-F12 medium containing fetal bovine serum (10%) and penicillin‒streptomycin (1%) solution in an incubator (37 °C, 5% CO_2_). After reaching confluence, the cells were detached with trypsin (0.25%) and passaged, and cells at passages 3–5 were used for the experiment. MAC-T cells were seeded in 6-well cell culture plates at a density of 2.0 × 10^4^ cells/cm^2^ and cultured for 24 h, after which the medium was replaced with complete DMEM-F12, and 20 µL of LPS (1 mg/mL) was then added. The control cells were treated with the same volume of PBS, and samples were collected after 3 h. Three biological replicates were established in each group, and the samples were stored at -80 °C.

### Total RNA extraction

Total RNA of BMECs was extracted according to the TRIzol reagent instructions. RNA integrity and the absence of gDNA contamination were verified by 1.0% denaturing agarose gel electrophoresis. The RNA concentration and purity in each sample were quantitatively measured by a NanoDrop ND-1000 spectrophotometer. The OD260/280 ratio was approximately 2.0. The RNA quality was assessed by an Agilent 2100 Bioanalyzer using an Agilent DNA 1000 chip kit, and the library was constructed only after the samples met the criteria for library construction.

### Library construction and sequencing

High-throughput mRNA and lncRNA sequencing and subsequent bioinformatics analysis were performed by Cloud-Seq Biotech (Shanghai, China). For each sample, 3.0 µg of RNA was treated with the NEBNext ® rRNA Removal Kit to remove rRNA. RNA sequencing libraries were constructed using the rRNA-depleted RNAs with the TruSeq Stranded Total RNA Library Prep Kit (Illumina, USA), and RNA was then reverse transcribed into cRNA. Quality control and quantification of the library were performed by using the BioAnalyzer 2100 instrument (Agilent Technologies, USA). After passing the inspection, the library was subjected to 150 bp paired-end sequencing on an Illumina NovaSeq system. Specifically, the Illumina NovaSeq 6000 system was used to sequence the library and generate paired-end reads.

### Data quality, sequence alignment and lncRNA identification

After paired-end sequencing with the Illumina NovaSeq 6000 system and acquisition of the raw reads, we used Q30 (the percentage of bases with an accuracy of 99.9% or higher) > 80% as the quality control standard for the original sequencing reads. Sequencing data contain some reads with adapters and of low quality. These sequences can greatly interfere with subsequent data analysis; thus, the sequencing data need further filtering. To filter our reads, we used Cutadapt software (v1.9.3) [47] to remove sequences with adapters at the 3’ end and reads with a base quality of less than 20 to obtain high-quality reads. The high-quality reads remaining after quality control were compared to the bovine reference genome (ARS-UCD1.2) with HISAT2 software (v2.0.4, http://ccb.jhu.edu/software/hisat2/index.shtml). We used HTSeq software (v0.9.1) to obtain the raw count of mRNA sequences at the transcript level and to generate mRNA expression profiles [48]. LncRNA sequences need to be aligned with those of the known lncRNAs in the National Center for Biotechnology Information (NCBI) lncRNA database. After successful alignment, we used HTSeq software (v0.9.1) to obtain the raw count of lncRNA sequences at the transcript level and to generate lncRNA expression profiles.

### Prediction of lncRNA target genes

Functional annotation of lncRNAs is accomplished primarily through bioinformatics analysis of lncRNA target genes, which facilitates the identification of the diverse biological functions and pathways in which they participate. The adjacent protein-coding genes within 100 kb upstream to 100 kb downstream of the lncRNAs were selected as their target genes from the NCBI database [49].

### Screening for DE lncRNAs and DE mRNAs

Using bioinformatics methods, the read count value for each gene was used as a measure of the baseline expression of the gene, and lncRNAs and mRNAs with statistically significant differences were then identified. The original counts in the LPS group and control group were normalized using trimmed mean of M values (TMM) normalization in edgeR software (v3.16.5) [50]. The difference between the two samples was calculated. The screening criteria for DE lncRNAs and DE mRNAs were as follows: fold change ≥ 2.0 and P ≤ 0.05.

### Functional enrichment analysis of DE lncRNAs and DE mRNAs

To explore the potential functions of the DE lncRNAs and DE mRNAs, GO (http://www.geneontology.org) and KEGG (http://www.kegg.jp) enrichment analyses were performed. Specifically, the KEGG database and GO database were used to annotate the DE genes and determine the number of DE genes. Pathways significantly enriched in the DE genes relative to the full genomic background were identified, Fisher’s exact test was applied, and the P values were calculated. For the main GO categories (biological process, cellular component and molecular function), the hypergeometric test was performed based on the GO term, and the enrichment score (-log_10_ (P value)) and P value were calculated. Only the KEGG pathways and GO terms with P ≤ 0.05 were considered markedly enriched. The hypergeometric test is the most common statistical method for enrichment analysis. In the equation below, N is the number of all genes in the specific organism that were annotated in a certain GO term (Background Genes), n is the number of DE genes annotated to the GO term, M is the number of all genes annotated to the GO Term (Pop Hit); and m is the number of DE genes annotated to the GO term (count):

$$\text{P}=1-\sum\limits_{i=0}^{{m-1}}\frac{\left(\genfrac{}{}{0pt}{}{M}{i}\right)\left(\genfrac{}{}{0pt}{}{N-M}{n-i}\right)}{\left(\genfrac{}{}{0pt}{}{N}{n}\right)}$$

### Construction of the mRNA–lncRNA coexpression network

A coexpression network was constructed using the DE lncRNAs and DE mRNAs to evaluate the relationships between the DE lncRNAs and DE mRNAs. This approach facilitates the identification of the pathways whose regulation involves the lncRNAs, thus allowing the prediction of the potential mechanisms of lncRNAs in disease. Pearson correlation coefficients between lncRNAs and mRNAs were calculated [51]. With |R| ≥ 0.90 and P < 0.01 as the threshold criteria, lncRNA‒mRNA relationship pairs were selected and visualized using Cytoscape software (v3.8.0) [52].

### Real-time fluorescence quantitative PCR

In this study, five DE lncRNAs and five DE mRNAs were randomly selected for qRT‒PCR validation of the high-throughput sequencing data. *β-Actin* served as the endogenous reference gene, and the primers specific for the selected genes were designed using Primer5 software and then tested with the NCBI Primer-Blast tool. The primer information is presented in Table 4. This assay was performed by the SYBR Green method using an Applied Biosystems Model 7500 Real-Time PCR system and a 96-well plate with the SYBR Green fluorescent dye kit from ABclonal. The reaction system consisted of 10 µL (1 µL template, 5 µL SYBR Green, 0.2 µL upstream primer, and 0.2 µL downstream primer; the remaining volume was supplemented with ddH2O to 10 µL). The thermal cycling program was as follows: 95 °C for 3 min, 95 °C for 5 s, and 60 °C for 34 s, for a total of 40 cycles. The relative expression levels of the selected DE genes were calculated using the 2-ΔΔCT method with three biological replicates per experiment and three technical replicates per reaction. All data from the experiment were analyzed and plotted in GraphPad Prism software (v8.0.2; Boston, Massachusetts, USA; www.graphpad.com).

**Table 4:** The primer sequences for qRT-PCR

Primer name	Primer sequence (5’→3’)	Fragment size (bp)
*LOC101904249*	F: TCTGCTATTAAGAAGCCACA	135
	R: CACGACTGAGCGACTGAC	
*LOC104976020*	F: TCTTTCAGGTTCGCCGTTTC	96
	R: GCTCGCCGCTTTGTTCAG	
*LOC101901950*	F: GCTCTGCCGAGAATGTGC	105
	R: GCGAGGCTTTGACTTGTGA	
*LOC104973139*	F: CCAGCACATCTGCCTAAT	103
	R: AAGACCCTGATCCTAACG	
*LOC107131253*	F: TGCCAGCCACCTCCTGTAGT	180
	R: GAGCCAGTTGTCCTCATCCTTT	
*TNFAIP3*	F: TTGGACCGATGAATGGGATA	130
	R: TGGCCTTCTGAGGATGTTGC	
*IL-1β*	F: CCTCCGACGAGTTTCTGTGT	85
	R: GCCAGCACCAGGGATTTTTG	
*IL-6*	F: ACTGGCAGAAAATAAGCTGAATCTTC	89
	R: TGATCAAGCAAATCGCCTGAT	
*BPGM*	F: CAGCCTTCCTTCTTGGGTAT	100
	R: TGGCATAGAGGTCTTTACGG	
*FOXL1*	F: CAATCTCGGCCCCATCAGAA	221
	R:CATCCCAAAAATGGGCAGGC	

### Statistical methods

Each experiment had at least three biological replicates and was repeated three times. GraphPad Prism 8.02 data processing software was used for statistical analysis, and one-way ANOVA was used to statistically analyze differences among the groups. The data in this experiment are expressed as the means ± SEMs.

## Data Availability

All data analyzed during this study are included in this published article. The raw data generated during the current study are available from the corresponding author upon reasonable request.

## List of abbreviations

LncRNA: Long noncoding RNA
DE: lncRNA differentially expressed lncRNA
DE mRNA: differentially expressed mRNA
BMEC: bovine mammary epithelial cell
LPS: lipopolysaccharide.
MAMP: main microbe-associated molecular pattern
TLR: Toll-like receptor
NLR: NOD-like receptor
NLRP3: NOD-like receptor family pyrin domain containing 3
RCD: regulated cell death
ROS: reactive oxygen species
